# MS‐Net: Learning to assess the malignant status of a lung nodule by a radiologist and her peers

**DOI:** 10.1002/acm2.13964

**Published:** 2023-03-16

**Authors:** Duwei Dai, Caixia Dong, Zongfang Li, Songhua Xu

**Affiliations:** ^1^ Institute of Medical Artificial Intelligence The Second Affiliated Hospital Xi'an Jiaotong University Xi'an Shaanxi China

**Keywords:** automatic features, empirical features, individual opinion, lung nodules, panel opinion

## Abstract

**Background:**

Automatically assessing the malignant status of lung nodules based on CTscan images can help reduce the workload of radiologists while improving their diagnostic accuracy.

**Purpose:**

Despite remarkable progress in the automatic diagnosis of pulmonary nodules by deep learning technologies, two significant problems remain outstanding. First, end‐to‐end deep learning solutions tend to neglect the empirical (semantic) features accumulated by radiologists and only rely on automatic features discovered by neural networks to provide the final diagnostic results, leading to questionable reliability, and interpretability. Second, inconsistent diagnosis between radiologists, a widely acknowledged phenomenon in clinical settings, is rarely examined and quantitatively explored by existing machine learning approaches. This paper solves these problems.

**Methods:**

We propose a novel deep neural network called MS‐Net, which comprises two sequential modules: A feature derivation and initial diagnosis module (FDID), followed by a diagnosis refinement module (DR). Specifically, to take advantage of accumulated empirical features and discovered automatic features, the FDID model of MS‐Net first derives a range of perceptible features and provides two initial diagnoses for lung nodules; then, these results are fed to the subsequent DR module to refine the diagnoses further. In addition, to fully consider the individual and panel diagnosis opinions, we propose a new loss function called collaborative loss, which can collaboratively optimize the individual and her peers’ opinions to provide a more accurate diagnosis.

**Results:**

We evaluate the performance of the proposed MS‐Net on the Lung Image Database Consortium image collection (LIDC‐IDRI). It achieves 92.4% of accuracy, 92.9% of sensitivity, and 92.0% of specificity when panel labels are the ground truth, which is superior to other state‐of‐the‐art diagnosis models. As a byproduct, the MS‐Net can automatically derive a range of semantic features of lung nodules, increasing the interpretability of the final diagnoses.

**Conclusions:**

The proposed MS‐Net can provide an automatic and accurate diagnosis of lung nodules, meeting the need for a reliable computer‐aided diagnosis system in clinical practice.

## 1 INTRODUCTION

Early detection and diagnosis are critical for preventing and treating lung cancers. Characterized by their high‐density resolutions, modern computed tomography (CT) images can reveal minute differences between normal anatomical structures and diseased tissues. For this reason, imaging technology has been widely adopted for screening lung cancers at an early stage.

In radiology, a lung nodule is diagnosed primarily according to its perceivable features, such as its grayscale, position, and morphology, in which the nodule's morphological^1^ features further include its texture,^2^ circumference, diameter, volume, compactness, roundness, curvature, and other perceptual features. Abundant prior studies^3^ show that burr‐shaped, rough, and leaf‐shaped lung nodules are more likely to be malignant, while nodules that exhibit smooth, round, oval, polygonal, or antennal shapes tend to be benign. However, these are the greatly simplified rule of thumb for diagnosis. In real‐world clinical practice, the manifestation of a malignant lung lesion on CT scans can be much more complicated and ambiguous, calling for deep radiological expertise to be acquired through years of clinical training, experience, and a superb level of human intelligence. Due to the steep demand for skills and the time‐consuming nature of lung cancer diagnosis based on CT scan reading, computer‐aided diagnosis (CAD)^4^, ^5^ of lung cancers has been keenly expected.

In response to such great demand, an assortment of efforts has been dedicated to the line of research for the past two decades, producing a collection of methods with impressive performances in diagnosing lung cancers from CT images.^6^, ^7^, ^8^ Despite these fruitful developments, two notable problems remain outstanding as follows: how to utilize perceptible empirical features for computer‐aided diagnosis? Computer‐aided diagnosis (CAD) system is a class of computer systems that aims to help the clinician to diagnose a disease. To construct such a lung nodule auxiliary diagnosis system, traditional CAD methods rely heavily on manual empirical features, such as nodule texture, curvature, and diameter.^9^, ^10^, ^11^ However, such empirical feature encoding operations are highly time‐consuming and unsystematic. As a result, traditional CAD methods obtain less than satisfactory performance, especially when put in comparison with modern deep learning‐based approaches. In contrast, existing deep learning‐based CAD methods (DL‐CAD)^12^ are usually architected as end‐to‐end trainable solutions, hence free from the aforesaid feature engineering problems. However, these end‐to‐end deep learning solutions tend to ignore the empirical features accumulated by radiologists through years of field practice and only rely on the features automatically discovered by the network to assess the malignant status of the lung nodules. This leads to the lack of interpretability and reliability for these solutions, which hinders their extensive application in clinical practice. How to cope with interobserver inconsistency while learning to diagnose lung nodules from multiple radiologists? Interobserver inconsistency is a well‐acknowledged problem in radiology.^13^, ^14^ To cope with contrasting malignancy ratings provided by a panel of radiologists concerning a nodule, existing approaches either treat these ratings as independent ratings cast over multiple unrelated nodules or merge these ratings through some voting, averaging, or other aggregation mechanisms into a single rating concerning the nodule. The former method neglects the diagnosis opinions of other experts in the panel, and the latter method erases individual diagnosis opinions. Both methods will lead to unreliable diagnosis results.

In recognition of the problems mentioned above, this study introduces a novel deep neural network (MS‐Net), which can make full use of the empirical features accumulated by radiologists through years of field practice and automatic features discovered by deep neural networks to provide accurate and reliable malignancy diagnosis for lung nodules. MS‐Net consists of two sequential modules, including a Feature Derivation and Initial Diagnosis module (FDID), followed by a Diagnosis Refinement module (DR). The FDID model of MS‐Net first derives a range of perceptible empirical features concerning a target nodule, which are used by radiologists to judge the malignancy of nodules, and provides two initial diagnoses, including individual diagnosis opinion and panel diagnosis opinion. Subsequently, the outputs of FDID are fed to the DR module to further refine the diagnoses. The outputs of the DR module are refined individual diagnosis opinions and panel diagnosis opinions. This design mechanism not only effectively uses perceptible empirical features and deep learning automatic features but also provides individual and panel diagnosis opinions on the malignant status of lung nodules, making the performance of the network better than other state‐of‐art deep learning algorithms and also increases the reliability and interpretability of the prediction results. To further strengthen the network's ability to learn both individual diagnostic opinions and panel diagnostic opinions simultaneously, we propose a new loss function called collaborative loss. Inspired by the label smoothing algorithm,^15^ collaborative loss softens individual opinion labels and panel opinion labels. When optimizing individual opinions, panel opinions will be applied as references. Similarly, individual opinions will be adopted as references when optimizing panel opinions. This loss function can coordinately optimize individual opinions and panel opinions to make up for each other's deficiencies, and further enhance the network's ability to assess the malignant status of lung nodules. Overall, the main contributions of this work are summarized as follows:

In summary, the main contributions of this work are three‐fold:

1. To take advantage of perceptible empirical (manual) features and deep learning automatic features to accurately and reliably assess the malignant status of lung nodules, we propose a novel network called MS‐Net, which comprises two sequential modules, including first a FDID, followed by a DR module.
2. To solve the inconsistent diagnosis of multiple radiologists concerning a nodule, we propose a new loss function called collaborative loss, which can collaboratively optimize individual and panel diagnosis opinions to provide more reliable diagnosis results.
3. Comprehensive experiments conducted on the LIDC database proved the effectiveness and reliability of MS‐Net for assessing the malignant status of lung nodules. Besides, as a byproduct, the new approach can automatically derive a range of perceptible nodule features, increasing the final diagnoses' interpretability.

## 2 DATASETS

### 2.1 Lung image database consortium dataset

The Lung Image Database Consortium image collection (LIDC‐IDRI) is a publicly available dataset.^16^ We use it to train and test the proposed methods. LIDC‐IDRI contains 1018 cases collected by seven institutions. Each case consists of at least one CT scan and associated XML file, recording nodule annotations made by up to four experienced radiologists. Each suspicious lesion is categorized as a non‐nodule, a nodule <3 mm, or a nodule ≥3 mm in diameter on the long axis. For nodules ≥3 mm, the XML file provides the corresponding nodule location, pixel‐level segmentation, malignancy likelihood, and eight semantic features (in this paper, we call it perceptible empirical features), including subtlety, internal structure, calcification, sphericity, margin, lobulation, spiculation, and texture.^16^ In XML files, the malignancy likelihood of nodules is rated from 1 to 5, indicating an increasing degree of malignancy suspiciousness; for example, a score of 1 means low malignancy, while a score of 5 indicates high malignancy (1–highly unlikely, 2–moderately unlikely, 3–indeterminate, 4–moderately suspicious, and 5–highly suspicious). Besides malignancy, most semantic features are also scored in the range of 1−5 in increasing order, while the internal structure and calcification are given scores in the range of 1−4 and 1−6, respectively.

### 2.2 Our usage of the LIDC‐IDRI dataset

#### 2.2.1 Extract nodule ROI

**FIGURE 1:**
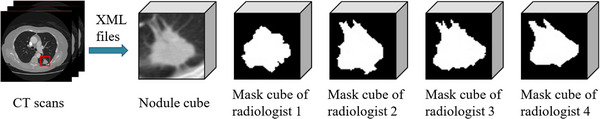
Extract nodule and mask cubes from CT images according to XML files.

The LIDC database contains a heterogeneous set of CT scans obtained using various acquisition and reconstruction parameters. To cope with the heterogeneity, we need to resample and normalize these scans before using them. First, we resample all scans to voxel spacing [0.68, 0.68, 1.75] using the nearest‐neighbor interpolation algorithm.^17^ The reason for using 1.75 mm as the slice spacing in the resampled CT data is that slice thicknesses vary from 0.6 to 5 mm, and their median is 1.75 mm. Then, we transform the resampled CT scans to Hounsfield (HU) scales [−1200, 600 HU], finally normalize them to a range of [0, 1].^18^ Since the maximum length of the whole nodules in the three dimensions is 57, 59, and 62, and the tissue surrounding the nodule is helpful in the diagnosis of the nodule, we crop an 80 × 80 × 80 mm cube (called nodule cube) region centered on the middle of the nodule centers provided by the corresponding XML file. Correspondingly, according to the corresponding XML file, we can also get 80 × 80 × 80 mm mask cubes offered by different radiologists (see Figure 1).

#### 2.2.2 Generate labels

Generally, the labeling method of nodules can be divided into two genres. The first genre treats the diagnostic opinion given by any radiologist as the nodule's ground truth label. We call such labels individual labels. In this case, if one nodule is annotated by four radiologists, it will be treated as four different nodules. The second genre utilizes the aggregated opinion of a panel of radiologists as the nodule's ground truth label. We call such labels panel labels. In this case, if one nodule is annotated by four radiologists, it is ground truth will be the average, or median of four radiologists' opinion. In this paper, individual labels and panel labels are generated as follows:

1. *Individual labels*: Only nodules labeled by at least three radiologists are selected, and CT scans with slice thickness larger than or equal to 3 mm are excluded. In this way, a total of 4252 nodule annotations are obtained. We label malignancy degrees 1, 2, and 3 as benign, and malignancy degrees 4 and 5 as malignant. Finally, we got 3212 benign nodules and 1040 malignant nodules.^19^
2. *Panel labels*: For nodules with an average score lower than 3, we label as them benign nodules; for those with an average score higher than 3, we label them as malignant nodules. We remove nodules with ambiguous IDs and those with an average score of 3, finally obtaining 880 benign and 495 malignant nodules.^7^
3. *Labels of eight semantic features*: For the calcification feature, we labeled level 1 to level 5 as one category, and level 6 as the other category. For the remaining seven features, we labeled level 1 to level 3 as one category, and level 4 and level 5 as another category.^19^

## 3 THE PROPOSED DEEP NEURAL NETWORK SOLUTION

### 3.1 Overview

**FIGURE 2:**
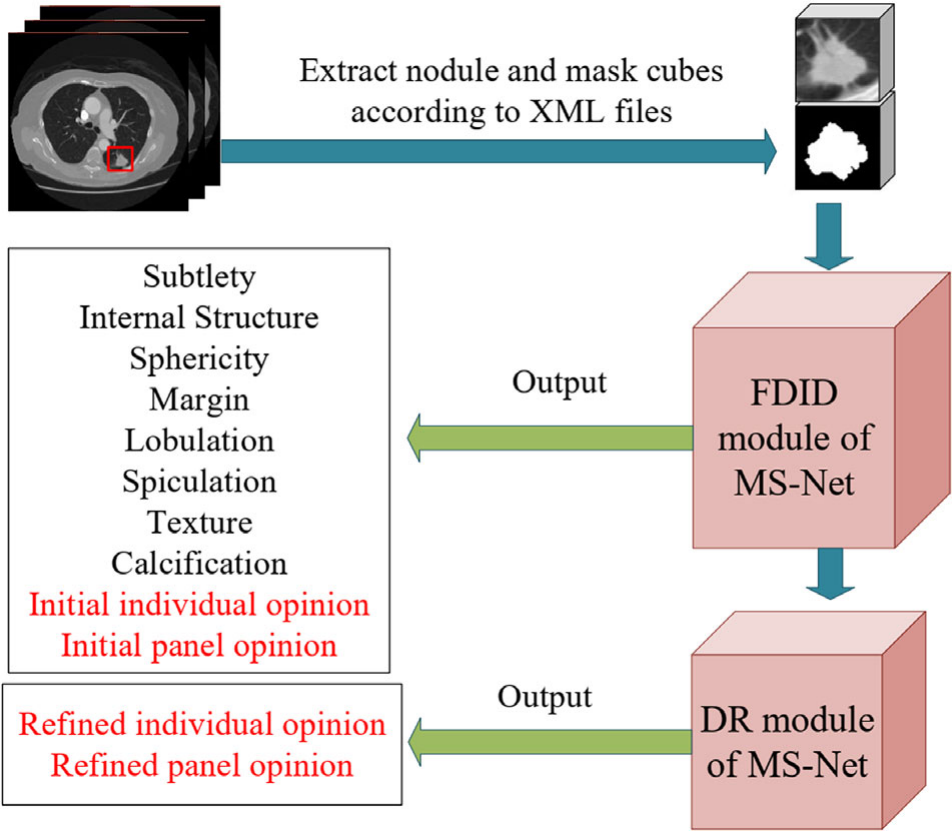
The flow chart of our MS‐Net to assess the malignant status of lung nodules. The output of the FDID module includes eight perceptible empirical features and two initial diagnoses, and the output of the DR module includes two refined diagnoses. DR, diagnosis refinement module; FDID, feature derivation and initial diagnosis module.

In this section, we will present the proposed MS‐Net for assessing the malignant status of lung nodules, whose workflow is shown in Figure 2.

The proposed deep neural network solution comprises two sequential modules: a FDID module, followed by a DR module.

For the first stage of the network (FDID), its inputs are concatenated nodule cubes and their corresponding mask cubes. The ground truth contains an individual label, a panel label, and eight empirical feature labels. The output is the prediction results of eight empirical features, including subtlety, internal structure, sphericity, margin, lobulation, spiculation, texture, and calcification, and two initial predictions of nodules, including individual and panel predictions. Note that the FDID predicts eight empirical features and two initial diagnoses simultaneously. This means that the diagnoses in the first stage are entirely data‐driven and do not utilize known clinical experience.

The reason for inputting the nodule cube and the mask cube at the same time is that the nodule cube contains the nodule's invariant information between different observers (for one particular nodule, its nodule cubes obtained by different radiologists are the same); the mask cube contains observer‐specific information (different radiologists usually draw different masks for one nodule, as shown in Figure 1). Since the network contains both the invariant information and the observer‐specific information of the particular nodule, and the ground truth includes both the individual and group labels, it can simulate multiple radiologists diagnosing the same nodule. When different radiologist diagnoses the same nodule, the network will output two predictions, one for the panel, and the other for the individual.

For the second stage of the network (DR), its inputs are the prediction result of eight empirical features and two data‐driven initial diagnosis results; its output is the refined diagnosis results. The design idea of the DR network is to optimize the data‐driven initial diagnosis based on clinical wisdom (eight empirical features).

### 3.2 The structure of the proposed MS‐Net

**FIGURE 3:**
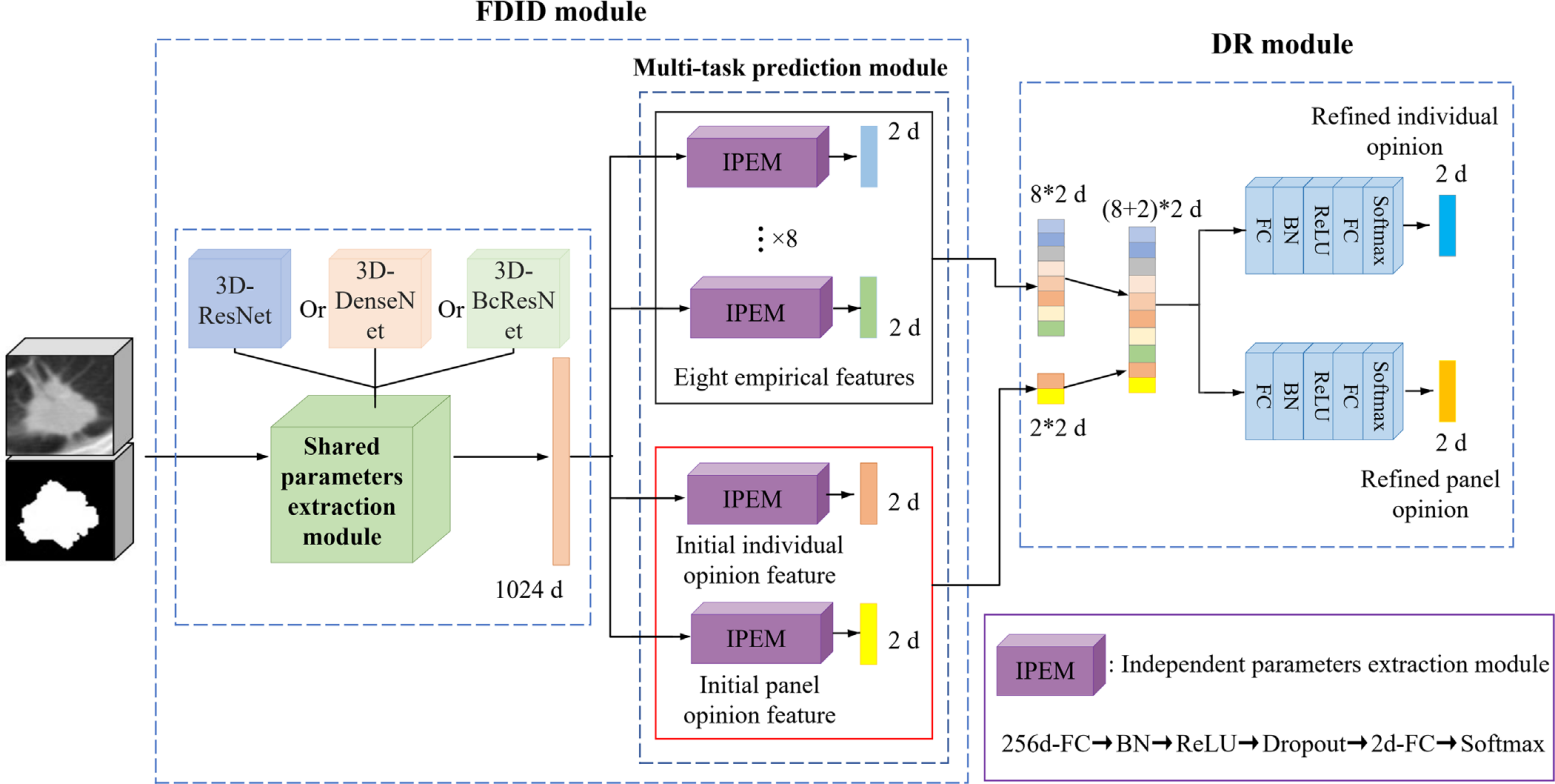
The architectural schema of the proposed MS‐Net. MS‐Net consists of two sequential modules, including a feature derivation and initial diagnosis module (FDID), followed by a diagnosis refinement module (DR). The FDID model first derives a range of perceptible empirical features and provides two initial diagnoses. Subsequently, the outputs of FDID are fed to the DR module to further refine the diagnoses. All diagnostic opinions are two‐dimensional, the first dimension represents the probability of benign, and the second represents the probability of malignant.

Figure 3 shows the structure of the proposed MS‐Net in detail. MS‐Net consists of two sequential modules: a FDID module, followed by a DR module.

#### 3.2.1 Feature derivation and initial diagnosis module

FDID mainly consists of a shared parameter extraction module and a multi‐task prediction module. The shared parameter extraction module extracts rich feature information from the nodule and mask cubes. Multi‐task prediction module is used to generate predictions for eight empirical features and two initial diagnoses.

**Shared parameter extraction module**: To comprehensively consider rich semantic relationships among imaging features and content captured by adjacent layers in a set of CT scans, we use a 3D convolutional neural network as the shared parameter extractor. The input of the shared parameter extractor is concatenated nodule cube and mask cube, and the output is a 1024‐dimensional feature (called shared feature).

**FIGURE 4:**
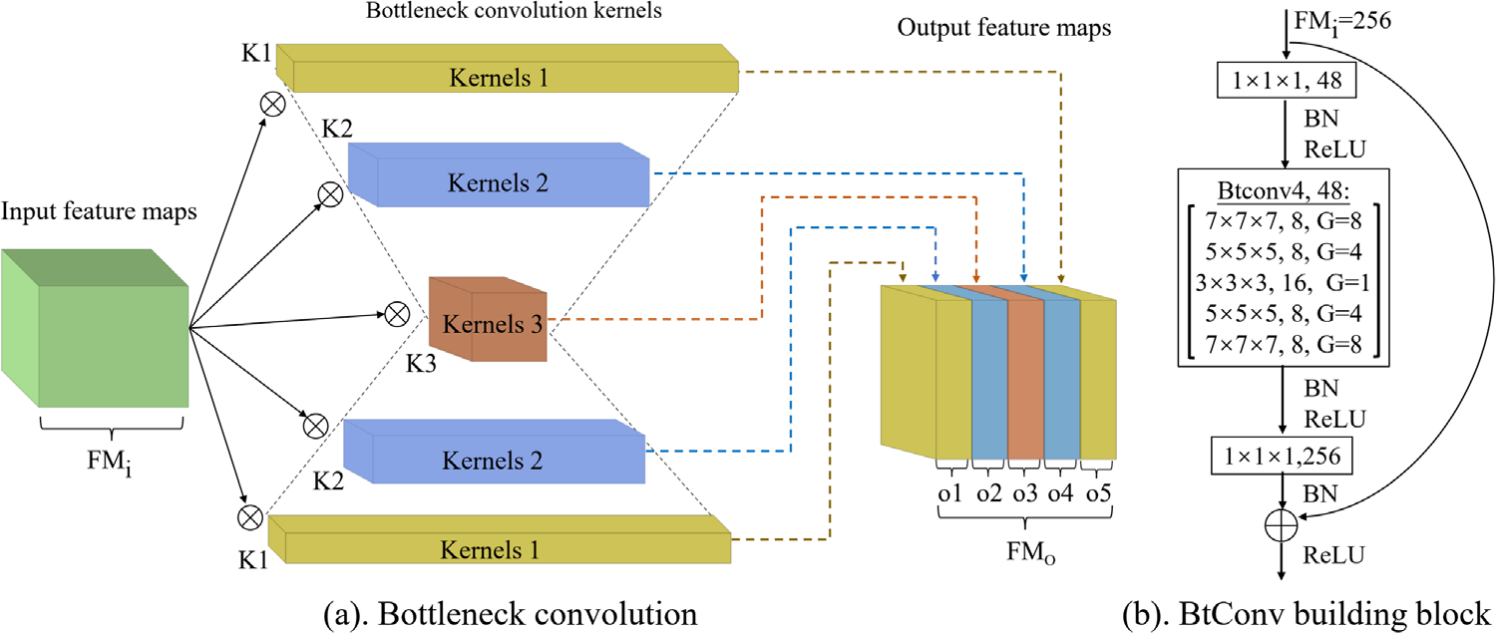
The internal composition of the bottleneck convolution and the BtConv building block built on it. In (a), k1, k2, and k3 represent convolution kernels with different sizes, respectively. In (b), G represents the number of groups in group convolutions.

The above‐mentioned 3D neural network can be popular 3D‐ResNet50,^20^ 3D‐DenseNet121,^21^ or our proposed 3D‐BcResNet50. Table 1 shows the construction details of 3D‐BcResNet50, which is mainly composed of the newly developed bottleneck convolution. Figure 4a schematically shows the internal composition of a bottleneck convolution structure, which consists of multiple parallel convolutions with different kernel sizes. The Figure 4b provides an example network building block based on a bottleneck convolution structure. As shown in Figure 4b, if the channel number of the input feature is 48, we first split it into five sub‐features along the channel axis; then, these sub‐features will go through convolution operations with different kernel sizes; finally, these five sub‐features are stacked together along the channel axis. These operations allow bottleneck convolutions to extract richer multi‐scale features than traditional convolutions.

**TABLE 1:** The construction details of the 3D‐BcResNet50.

Stage	Output	3D‐ResNet50	3D‐BcResNet50
	80×80×80	7×7×7 Stride (2, 2, 2)	7×7×7 Stride (2, 2, 2)
1	40×40×40	$\left[\begin{array}{c} 1\times 1\times 1,\;64\\ 3\times 3\times 3,\;64\\ 1\times 1\times 1,\;256\; \end{array}\right]\times 3$	$\left(\begin{array}{c} 1\times 1\times 1,\;48\\ BConv4,\;48:\\ \left[\begin{array}{c} 7\times 7\times 7,\;8,\;G\;=\;8\\ 5\times 5\times 5,\;8,\;G\;=\;4\\ \;3\times 3\times 3,\;16,\;G\;=\;1\\ 5\times 5\times 5,\;8,\;G\;=\;4\\ 7\times 7\times 7,\;8,\;G\;=\;8 \end{array}\right]\\ 1\times 1\times 1,\;192 \end{array}\right)\times 3$
2	20×20×20	$\left[\begin{array}{c} 1\times 1\times 1,\;128\\ 3\times 3\times 3,\;128\\ \;1\times 1\times 1,\;512\; \end{array}\right]\times 4$	$\left(\begin{array}{c} 1\times 1\times 1,\;96\\ BConv3,\;96:\\ \left[\begin{array}{c} 7\times 7\times 7,\;16,\;G\;=\;8\\ 5\times 5\times 5,\;16,\;G\;=\;4\\ \;3\times 3\times 3,\;32,\;G\;=\;1\\ 5\times 5\times 5,\;16,\;G\;=\;4\\ 7\times 7\times 7,\;16,\;G\;=\;8 \end{array}\right]\\ 1\times 1\times 1,\;384 \end{array}\right)\times 4$
3	10×10×10	$\left[\begin{array}{c} 1\times 1\times 1,\;256\\ 3\times 3\times 3,\;256\\ \;1\times 1\times 1,\;1024\; \end{array}\right]\times 6$	$\left(\begin{array}{c} 1\times 1\times 1,\;192\\ BConv2,\;192:\\ \left[\begin{array}{c} 5\times 5\times 5,\;48,\;G\;=\;4\\ 3\times 3\times 3,\;96,\;G\;=\;1\\ 5\times 5\times 5,\;48,\;G\;=\;4 \end{array}\right]\\ 1\times 1\times 1,\;768 \end{array}\right)\times 6$
4	5×5×5	$\left[\begin{array}{c} 1\times 1\times 1,\;512\\ 3\times 3\times 3,\;512\\ \;1\times 1\times 1,\;2048\; \end{array}\right]\times 3$	$\left(\begin{array}{c} 1\times 1\times 1,\;384\\ BConv1,\;384:\\ \left[3\times 3\times 3,\;384,\;G\;=\;1\right]\\ 1\times 1\times 1,\;1536 \end{array}\right)\times 3$
	1×1×1	Global avg pool 1024‐d fc	Global avg pool 1024‐d fc

**Multi‐task prediction module**: The input of the multi‐task prediction module is the above‐mentioned 1024‐dimensional shared features, and the output is the prediction of eight empirical features and the prediction of two initial diagnoses. The multi‐task prediction module consists of 10 parallel branches. In each branch, the 1024‐d shared features undergo fully connected layers (FC, 1024‐d–256‐d), batch normalization (BN), ReLU, Dropout (ratio = 0.5), FC (256‐d–2‐d), and Softmax^22^ operations to generate a 2‐d prediction probability. Since the activation function is Softmax, according to the calculation formula of Softmax, the sum of the probabilities of 2‐d prediction is 1. This module predicts eight empirical features and two initial diagnoses simultaneously. Therefore, the initial diagnoses are entirely data‐driven and do not utilize known clinical experience.

#### 3.2.2 Diagnosis refinement module

This module aims to refine two initial diagnoses using eight empirical features. Its input is the prediction of eight empirical features and two initial diagnoses, and the output is the refined individual diagnosis and panel diagnosis. The refined individual and panel diagnosis are both a 2‐dimension feature; its first dimension represents the probability of benign, the second dimension represents the probability of malignancy, and the sum of the two is 1. The specific operation of this module is as follows: first, concatenate eight empirical features and two initial diagnoses to obtain a fusion feature; then, the fusion feature goes through FC, BN, ReLU, FC, and Softmax operations to generate the refined individual diagnosis and panel diagnosis, respectively.

### 3.3 The proposed loss function

It is often that different radiologists may have an inconsistent diagnosis of the malignant status concerning the same nodule. To ensure the independence and reliability of the radiologist's diagnosis, we propose a novel loss function called collaborative loss. It can mediate between the diagnostic opinion of a given radiologist and the panel opinion from multiple peer radiologists.

Concerning the common classification problems, usually Softmax^22^ or Sigmoid^23^ is processed first and cross‐entropy calculation is performed after. The calculation of loss can be understood as a punishment rule to make the probability distribution of the data prediction close to its true distribution. One‐hot^24^ is usually adopted to encode real labels. In the classification of the malignant status of nodules, the one‐hot encoding method will cause the model to over‐trust the current radiologist's diagnosis opinions (individual labels), and completely neglect the diagnosis opinions of her peers (panel labels). To solve the above problem, we consider softening this encoding method.^15^, ^25^ After the coding method is softened, when the loss function evaluates whether the prediction result of the neural network model is correct, it considers not only the individual label but also the panel label. We use the following formula to represent the label distribution after softening:

(1) $$q^{\prime }\left(k|x\right)\;=\;\left(1-\varepsilon \right)\delta_{k,y_{0}}+\varepsilon \theta_{k,y_{1}}$$

in formula (1), *k* represents a specific type of nodule: 1 represents benign, and 2 represents malignant; *y*_0_ means individual label, *y*_1_ means panel label; 𝛿 represents the distribution of the individual labels; 𝜃 represents the distribution of the panel labels; 𝜀 is the weight of $\theta_{k,y_{1}}$, which in the range of [0, 1] and defaulted to 0.6 in our study; (1−𝜀) is the weight of $\delta_{k,y_{0}}$; $q^{\prime }$ is the label distribution after softening. In this case, if the individual label is 1 and the group label is 1, the soft label will be encoded as [1,0]; if the individual label is 1 and the group label is 2, the soft label will be encoded as [0.4, 0.6].

We replace the one hot encoding method in the traditional cross‐entropy^26^ loss function with the soft label encoding method to obtain our collaborative loss ($L_{cb}$):

(2) $$\begin{array}{ccc} L_{cb}\;\left(q^{\prime },p\right) & = & \;-\;\sum_{k\;=\;1}^{K}\log p\left(k\right)q^{\prime }\left(k\right)\\ & = & \;\left(1-\varepsilon \right)H\left(q_{0},p\right)+\varepsilon H\left(q_{1},p\right) \end{array}$$

in formula (2), $q^{\prime }$ represents the softened label, and *p* represents the distribution predicted by the network; K is the total number of classes (in this paper, *K* is 2), k∈[1,K].

### 3.4 Training strategy and model evaluation

#### 3.4.1 Data augmentation

To alleviate model overfitting, we perform data augmentations, including random image translation, rotation or flip operation, to each sample in the training set online. The translation step is selected from [0, 5] voxels; the rotation is done by first swapping the three axes in 3‐D followed by a 2‐D rotation of [45°, 135°, 225°]; the flip operation is performed on all three axes.^7^ In the meantime, we perform more data augmentations on malignant nodules than benign ones to balance the total number of benign and malignant lung nodules.

#### 3.4.2 Experiment details

The proposed MS‐Net is implemented in the PyTorch library with an NVIDIA 2080ti GPU (11G onboard memory). We use the Xavier^27^ to initialize the model's learnable parameters. We employ stochastic gradient descent (SGD) as the overall optimizer for updating all parameters, and the batch size in training is set to eight. Since our proposed solution contains two modules, the first module FDID and the second module DR, and the result of the second module depends on the output of the first module, given this, we divided the training of the neural network into two steps. In the first step, we only trained the parameters in the FDID module of the network. After 40 epochs, train the FDID module and DR module together, and the network is trained for a total of 80 epochs. It is worth noting that the learning rate is set to 0.01 in the first stage and decreased to 0.0001 in the second stage due to the less amount of learnable parameters in the DR module.

#### 3.4.3 Evaluation metrics

In this study, the model's performance is assessed by accuracy, sensitivity/recall, specificity, precision, and F1‐score metric. Accuracy shows the performance of the proposed method in classifying nodules as malignant or benign. Sensitivity and specificity measure the proportion of malignant and benign nodules that are correctly identified, respectively. Precision is the fraction of retrieved true positive instances among the retrieved positive instances. The F1‐score is a measure of a test's accuracy and considers precision and recall.

## 4 EXPERIMENT

To explore the performance of the proposed MS‐Net for assessing the malignancy status of a lung nodule, we compared it with eight state‐of‐art deep learning models. Besides, we compare the performance of different shared parameter extraction methods and loss functions in the proposed MS‐Net system. And, the specific settings are as follows:

1. **Model1**: 3D‐ResNet50 + cross‐entropy. Namely, use 3D‐ResNet50 as the shared parameter extractor and cross‐entropy loss function as the optimizer.
2. **Model2**: 3D‐DenseNet121 + cross‐entropy.
3. **Model3**: 3D‐BcResNet50 + cross entropy.
4. **Model4 (MS‐Net)**: 3D‐BcResNet50 + collaborative loss.

To ensure the fairness of comparison, all methods are trained and tested using the same training and test data set, where the labeling method for each case is also standardized. As we described in Section 2.2, there are two primary methods of nodule labeling: individual labels and group labels. Therefore, in our experiments, we conduct performance benchmarking and comparison using the two labeling methods in two separate experimental rounds.

### 4.1 Comparative experiment using panel labels as the ground truth

Hua et al.,^28^ Shen et al.,^7^, ^19^ Xie et al.,^29^ and Xu et al.^30^ use panel labels as the ground truth when assessing the malignancy of nodules. In this way of labeling, we finally obtain 880 benign and 495 malignant nodules. We employ 825 nodules (528 benign and 297 malignant) in the training set, 275 nodules (176 benign and 99 malignant) in the validation or test set, and perform five‐fold cross‐validation. Table 2 shows the performance of different models using panel labels as ground truth. In Table 2, the performances of Hua et al.,^28^ Shen et al.,^7^ and Shen et al.^19^ are taken from their articles, and the performances of Xie et al.^29^ and Xu et al.^30^ are reproduced by us.

**TABLE 2:** The performance of the methods using panel labels as ground truth.

Methods	Number	Accuracy	Sensitivity	Specificity	Precision	F1‐score
Hua et al.^28^	2545	–	73.7	78.7	–	–
Shen et al.^19^	1375	84.0	–	–	–	–
Shen et al.^7^	1375	87.1	–	–	–	–
Xie et al.^29^	1375	88.0 ± 0.53	88.9 ± 0.46	87.5 ± 0.35	80.0 ± 0.38	84.2 ± 0.49
Xu et al.^30^	1375	90.9 ± 0.58	89.9 ± 0.47	91.5 ± 0.39	85.6 ± 0.37	87.7 ± 0.52
Model1	1375	89.5 ± 0.4	90.9 ± 0.33	88.6 ± 0.32	81.8 ± 0.35	86.1 ± 0.50
Model2	1375	90.5 ± 0.51	91.9 ± 0.40	89.7 ± 0.34	83.5 ± 0.41	87.5 ± 0.47
Model3	1375	91.6 ± 0.46	91.9 ± 0.36	91.5 ± 0.37	85.8 ± 0.39	88.8 ± 0.44
Model4	1375	**92.4 ± 0.54**	**92.9 ± 0.41**	**92.0 ± 0.35**	**86.8 ± 0.43**	**89.8 ± 0.51**

The best results are shown in bold.

As can be seen from Table 2, the accuracy of our proposed MS‐Net (Model 4) is 92.4%, 4.4% higher than Xie et al.^29^ and 1.5% higher than Xu et al.,^30^ which proves the effectiveness of MS‐Net in the diagnosis of lung nodules. Compared with Model1 and Model2, the accuracy of Model3 has increased by 2.1% and 1.1%, respectively, which shows that our proposed 3D‐BcResNet50 is superior to 3D‐ResNet50 and 3D‐DenseNet121 in extracting valuable features from CT images to help improve lung nodule diagnosis. Comparing Model3 and Model4, it can be seen that thanks to collaborative loss, the accuracy of the model has increased by 1.2%, which shows that collaboratively study individual diagnosis opinions and panel diagnosis opinions can help improve individual diagnosis.

### 4.2 Comparative experiment using individual labels as the ground truth

Kumar et al.,^31^ Song et al.,^8^ and Shen et al.^19^ adopt individual labels as the ground truth when assessing the malignancy of nodules. In this way of labeling, we finally get 3212 benign nodules and 1040 malignant nodules. We split these samples into four subsets, where each subset has a similar number of nodules. Two subsets are used for training, one subset for validation, and one subset for testing. Table 3 shows the performance of different models using individual labels as ground truth. In Table 3, the performances of Kumar et al.,^31^ Song et al.,^8^ and Shen et al.^19^ are taken from their articles, and the performances of Xie et al.^29^ and Xu et al.^30^ are reproduced by us.

**TABLE 3:** The performance of the methods using individual labels as ground truth.

Methods	Number	Accuracy	Sensitivity	Specificity	Precision	F1‐score
Kumar et al.^31^	4323	75.0	–	–	–	–
Song et al.^8^	5024	84.2	–	–	–	–
Shen et al.^19^	4252	84.3	70.5	88.9	–	–
Xie et al.^29^	4252	86.1 ± 0.77	72.7 ± 0.58	90.3 ± 0.46	70.4 ± 0.54	71.5 ± 0.55
Xu et al.^30^	4252	87.3 ± 0.63	77.0 ± 0.51	90.6 ± 0.44	72.1 ± 0.49	74.5 ± 0.53
Model1	4252	86.2 ± 0.47	74.2 ± 0.38	90.0 ± 0.35	70.1 ± 0.41	72.1 ± 0.47
Model2	4252	87.1 ± 0.55	76.6 ± 0.42	90.5 ± 0.38	71.8 ± 0.43	74.1 ± 0.50
Model3	4252	87.7 ± 0.51	77.3 ± 0.40	91.1 ± 0.37	73.3 ± 0.43	75.3 ± 0.51
Model4	4252	**88.5 ± 0.57**	**78.1 ± 0.44**	**91.8 ± 0.40**	**75.2 ± 0.44**	**76.6 ± 0.53**

The best results are shown in bold.

It can be seen from Table 3 that the accuracy of our proposed MS‐Net (Model4) can reach 88.5%, which is 4.2%, 2.4%, 1.2% higher than Shen et al.,^19^ Xie et al.,^29^ and Xu et al.^30^ respectively, which once again proves that MS‐Net can accurately and reliably diagnose lung nodules. Thanks to the stronger feature extraction capabilities of 3DBcResNet50 than 3D‐ResNet50 and 3D‐DenseNet121, the accuracy of Model3 is 1.5% and 0.6% higher than Model1 and Model2, respectively. The accuracy of Model4 is 0.8% higher than Model3, which once again confirms the role of collaborative loss in improving the accuracy of nodule diagnosis. It is worth noting that the accuracy of Model4 in Table 2 is 0.924, and in Table 3 is 0.885. We consider that when individual diagnosis labels are used as ground truth, there is much inconsistency in the diagnosis concerning the same nodule, which can lead to decreased accuracy of the model.

### 4.3 Coexistence of individual and panel labels

We argue that it is not appropriate to merge the labels of the same nodule or treat them separately. The merger will erase the independent judgment of the radiologist, and separate treatment neglects the judgment of other radiologists, leading to inaccurate and unreliable diagnoses. Therefore, the proposed MS‐Net and collaborative loss provide both the individual diagnosis opinion and the expert panel diagnosis opinion. We evaluate the diagnostic performance of MS‐Net on 4252 nodules and perform four‐fold cross‐validation. The performance is shown in Table 4.

**TABLE 4:** The performance of MS‐Net on coexisting individual labels and panel labels.

Attributes	Accuracy	Sensitivity	Specificity	Precision	F1‐score
FDID individual opinion	87.2 ± 0.49	76.6 ± 0.36	90.6 ± 0.38	72.1 ± 0.46	74.2 ± 0.47
DR individual opinion	88.3 ± 0.45	78.1 ± 0.35	91.6 ± 0.36	74.6 ± 0.43	76.3 ± 0.44
FDID panel opinion	91.4 ± 0.52	85.2 ± 0.40	93.6 ± 0.39	81.9 ± 0.47	83.5 ± 0.50
DR panel opinion	**92.2 ± 0.43**	**86.7 ± 0.34**	**94.1 ± 0.35**	**83.3 ± 0.43**	**85.0 ± 0.46**

The best results are shown in bold. DR, diagnosis refinement module; FDID, feature derivation and initial diagnosis module.

The results in Table 4 show the inconsistency between individual and panel opinions, proving the necessity of simultaneously outputting both types of opinions. In addition, DR results are better than FDID, demonstrating the effectiveness of our proposed diagnosis refinement module.

### 4.4 Performance on different number of training samples

**FIGURE 5:**
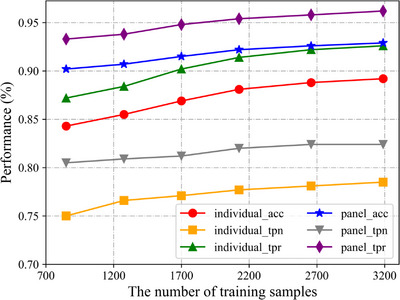
The performance of MS‐Net on different number of training samples.

Deep neural networks usually require a large amount of labeled data to achieve good performance, however, it is rather difficult to acquire labeled data in the medical field. To evaluate the dependence of MS‐Net on training data, we fed a different number of samples to it. This experiment was still based on the 4252 nodule annotations above‐mentioned, and we selected 1063 nodular annotations as the test set. The performance of MS‐Net on the different numbers of training samples is shown in Figure 5.

It can be seen from Figure 5 that with the reduction of training data, the performance of the model has a slight decline, and even if only 800 data are applied for training (1063 in the test), MS‐Net still has good performance, which proves that MS‐Net has low data dependency.

#### 4.4.1 Performance on multiple empirical features

**FIGURE 6:**
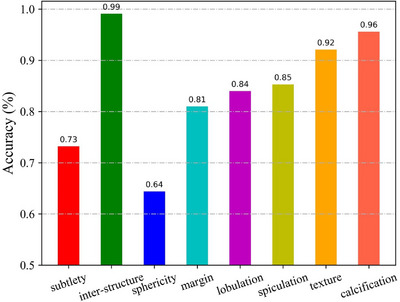
The performance of MS‐Net on predicting multiple empirical features.

As an intermediate process of MS‐Net, the FDID module can provide eight empirical features of lung nodules, including subtlety, internal structure, sphericity, margin, lobulation, spiculation, texture, and calcification, which have been clinically proven to be helpful for assessing the malignancy of lung nodules. Figure 6 shows the performance of MS‐Net in predicting multiple perceptible features. It can be seen from Figure 6 that MS‐Net can accurately predict the most empirical features of lung nodules, which increases the interpretability of the model. However, the predictions for subtlety and sphericity are unsatisfactory, and further improvement is needed.

**FIGURE 7:**
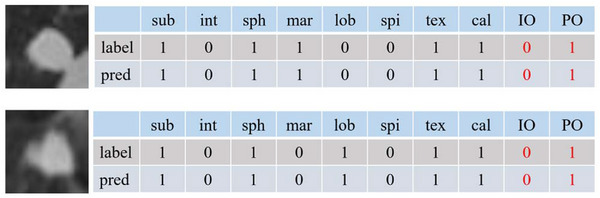
Predicted results of multiple perceptible features and malignant status concerning two lung nodules. IO stands for individual diagnosis opinion, PO stands for panel diagnosis opinion. Other features' names are replaced with their first three letters.

Figure 7 qualitatively shows the prediction performance of MS‐Net on lung nodules. It can be seen that MS‐Net can provide accurate diagnoses even if the individual opinions are inconsistent with the panel opinions.

## 5 LIMITATIONS AND FUTURE WORKS

Although the proposed MS‐Net improves the accuracy and reliability of lung nodule diagnosis, there is still room for improvement. We consider the key to progress lies in increasing the amount of training data and balancing the number of benign and malignant nodules. Considering that it is difficult to obtain and label enough medical images, we plan to use the generative adversarial network (GAN) to generate more nodule samples in future work. We will generate specific nodules under the guidance of perceptible features. Once there is sufficient data, a better‐performing network can be expected. In addition, we will also validate and improve the model's generalization ability in multiple medical centers.

## 6 CONCLUSIONS

In this study, we proposed a novel deep‐learning‐based model (MS‐Net) for assessing the malignant status of a lung nodule, which comprises two sequential modules, including first a FDID module, followed by a DR module. The FDID model of MS‐Net first derives a range of empirical features and provides initial diagnoses for lung nodules, then these results are fed to the subsequent DR module to further refine the diagnoses. Through this design, MS‐Net can combine the advantages of perceptible empirical features and deep learning automatic features to provide more reliable diagnoses for lung nodules. To strengthen the learning‐to‐diagnosis capability of MS‐Net, we equipped it with a newly proposed deep learning backbone (3D‐BcResNet50), which has a stronger ability to extract rich multi‐scale features than 3D‐ResNet50 and 3D‐DenseNet121, and a newly designed collaborative loss function, which can jointly learn from both individual radiologists and a panel of peer readers. Comprehensive experimental results convincingly demonstrated that MS‐Net significantly outperforms eight state‐of‐the‐art peer methods in diagnosing lung nodule malignancy. As a byproduct, the new approach can automatically derive a range of empirical features of lung nodules, which can increase the interpretability of the final diagnosis. All of these confirm that MS‐Net has excellent potential in the computer‐aided diagnosis of lung nodules.

## AUTHOR CONTRIBUTIONS

Study design, data analysis, and manuscript drafting: Duwei Dai and Caixia Dong. Manuscript revision: Zongfang Li. Study guidance, manuscript revision, and financial support: Songhua Xu.

## CONFLICT OF INTEREST STATEMENT

The authors declare no conflict of interest.
